# Staying Safe: lessons from suicide prevention for chiropractors and osteopaths

**DOI:** 10.1186/s12998-025-00607-x

**Published:** 2025-10-21

**Authors:** Stanley Innes, Jean Théroux, Judith Hope

**Affiliations:** 1 Adult Mental Health and Wellbeing Program, Eastern Health, Melbourne, Australia; 2 Eastern Health Clinical School, Monash University, Melbourne, Australia; 3 CHU Sainte-Justine Hospital, Montreal, Canada; 4 Centre for Mental Health Education and Research, Delmont Private Hospital, Melbourne, Australia

**Keywords:** Suicide prevention, Patient safety, Chiropractors, Osteopaths, Biopsychosocial approach, Professional-patient relations

## Abstract

Suicide remains a major cause of preventable death worldwide. A recent guidance document from NHS England (*Staying Safe from Suicide*, 2025) highlights the limitations of traditional suicide risk prediction methods and advocates for a relational, person-centred approach. While not mental health specialists, chiropractors and osteopaths often work closely with individuals facing musculoskeletal chronic pain, disability, financial stress, and social isolation, all of which are risk factors for psychological distress. This commentary explores how the NHS guidance offers key lessons for chiropractic and osteopathic practice. Valuable contributions to suicide prevention efforts can be made by fostering strong therapeutic relationships, adopting a biopsychosocial view of health, and encouraging help-seeking behaviours where needed. Through small, relational actions, practitioners can support patient wellbeing while working within the boundaries of their professional scope of practice.

**Supplementary Information:**

The online version contains supplementary material available at 10.1186/s12998-025-00607-x.

## Introduction

Suicide remains a leading cause of preventable death globally, with rates rising across many populations [1]. Traditionally seen as the domain of psychiatry, psychology, and mental health services, suicide prevention is now recognised as a shared responsibility across the broader healthcare system [2]. While there have been recent commentaries exploring and supporting physical therapists [3, 4] and podiatrists [5, 6] to this end, little has been undertaken for other musculoskeletal practitioners [7].

Chiropractors and osteopaths are trusted health professionals who provide regular care and are well-positioned to contribute to this effort [8]. Many people who consult with chiropractors and osteopaths live with chronic pain, disability, social isolation, financial hardship, or mental health vulnerabilities, all of which are established risk factors for suicide [9]. Almost one quarter of people with painful comorbidities have experienced suicidal ideation during their lifetime [10].

The National Health Scheme (NHS) is a publicly funded healthcare system that provides universal healthcare to all UK residents. In 2025, the NHS released *Staying Safe from Suicide*, a new guidance document intended to transform how healthcare providers approach safety assessment and care for individuals at risk [11]. The guidance firmly rejects traditional suicide risk prediction tools and categorisations, noting that risk fluctuates rapidly and cannot be reliably assessed through static means [12]. Instead, it advocates for a relational, person-centred, and biopsychosocial approach that focuses on understanding each individual’s circumstances, fostering hope, and supporting safety collaboratively [12].

This commentary seeks to explore how the key messages from *Staying Safe from Suicide* are relevant to chiropractic and osteopathic practice. It argues that adopting a relational mindset, using compassionate communication, and understanding biopsychosocial influences on wellbeing are not only consistent with a holistic approach to chiropractic and osteopathy but can also enhance health care more broadly. Without requiring such practitioners to act outside their scope of practice, small changes in approach can positively impact patient safety and overall experience.

## Discussion

The NHS England guidance *Staying Safe from Suicide* (2025) challenges several long-held assumptions about suicide risk assessment in healthcare [11]. Although primarily aimed at mental health services, its core messages have relevance for all healthcare providers. Five important principles and seven practical suggestions emerge and are summarized in Table 1.

**Table 1:** Summary of 5 key principles and 7 practical suicide prevention strategies for chiropractors and osteopaths from the NHS Staying Safe guidelines

Principle	Key message	Practical application
Risk prediction is unreliable	Traditional "low-medium–high" suicide risk labels are flawed and unhelpful	Focus on the individual’s experience and signs of distress instead of relying on static or tick box tools / questionnaires
Relational safety is crucial	A warm, compassionate therapeutic relationship improves safety and clinical outcomes	Foster trust by listening, validating feelings, and avoiding judgment
Biopsychosocial lens is essential	Suicide risk is influenced by biological, psychological, social factors, and not just physical symptoms	Consider how pain, isolation, trauma, or financial stress may impact a patient’s wellbeing and outlook
Risk is dynamic, not static	A person’s risk can change quickly. One assessment cannot guarantee future safety	Stay alert to behavioural or mood changes and check in regularly where appropriate
Responding safely and collaboratively	Don’t rely on tick-box risk assessments. Practitioners are encouraged to work collaboratively with patients to enable the identification of warning signs that leads to sources of support	Referring to a mental healthcare in order for the healthcare consumer to co-create with them a personalised safety plan via conversation rather than form-filling that empowers the individual and respects their autonomy

Practical strategies	Key message	Practical application
Recognising distress	Subtle signs like withdrawal, hopeless statements, or unusual silence may signal emotional pain	Gently explore signs of distress without assuming or diagnosing
Empathic engagement	Compassionate, curious questions reduce stigma and foster connection	Ask, “How have you been coping?” instead of checklist-style queries
Use open-ended communication		
Support-seeking	Patients benefit when help-seeking is normalised and easy to access	Encourage follow-up with a GP or psychologist if needed
Referrals	Know local mental health and crisis referral options	Provide information when necessary
Sensitive documentation	Avoid speculative statements about suicide risk	Record patient quotes or behaviours objectively (e.g., “Patient said, ‘There’s no point anymore’”) and notify other professionals where necessary
Practitioner wellbeing	Emotional impact is real and can be heightened in those with lived experience	Seek supervision or peer support when needed. Caring for yourself enables better care for others

### Risk prediction is unreliable

Traditional methods of predicting suicide risk, such as the Suicide Risk Scales (SRS) [13], categorize an individual’s risk as "low," "medium," or "high" are inherently flawed [14, 15]. Research shows that many people who die by suicide after contact with services were previously assessed as "low risk"[16]. Suicidal thoughts can fluctuate rapidly, making static assessment unreliable by nature. Rather than attempting to predict risk, healthcare practitioners are encouraged to focus on understanding the person's lived experience and current safety concerns.

### Relational safety is crucial

The *Staying Safe from Suicide* initiative seeks to move away from protecting against suicide using assessment strategies, such as a checklist approach, towards an understanding approach facilitated by the therapeutic relationship. Relational safety enhances understanding and increases disclosure and help-seeking. The building of a warm, compassionate, and trusting connection with patients improves safety and clinical outcomes [17]. Practitioners are encouraged to engage openly, validate distress, and create a safe space where patients can share their experiences without fear of judgment.

### A biopsychosocial understanding is essential

Suicidal distress arises within a complex interplay of biological, psychological, and social factors. A purely physical or symptom-focused approach may overlook important contributors to a person's risk or suffering. Adopting a biopsychosocial lens that involves exploring aspects such as musculoskeletal issues in terms of pain, disability, social isolation, financial stress, and psychological resilience allows for a more comprehensive understanding of a patient's pain, wellbeing, recovery and overall health outcomes [18].

Importantly, this biopsychosocial view should also incorporate the social determinants of health. Where a person is born, grows, lives, works, worships, and plays profoundly shapes their opportunities, stressors, and overall wellbeing. These contextual drivers—such as housing stability, access to education and healthcare, employment conditions, cultural supports, and community safety—intertwine with biological and psychological factors. For chiropractors and osteopaths, this means remaining attuned not only to the presenting musculoskeletal concern but also to the wider social context that may amplify or mitigate distress and recovery.

### Risk is dynamic, not static

Suicidal risk can shift within minutes or hours. An assessment carried out during one clinical encounter cannot guarantee safety in the future. Where appropriate, regular, informal check-ins about a patient’s wellbeing, along with remaining attentive to changes in behaviour, mood, or life circumstances, are more valuable than relying on past assessments alone.

### Safety planning is collaborative, not prescriptive

Rather than relying on the use of tick-box risk assessments, practitioners are encouraged to work collaboratively with patients to identify warning signs, coping strategies, and sources of support. A personalised, co-created safety plan with a mental healthcare practitioner developed through conversation rather than form-filling empowers the individual and respects their autonomy. Importantly, involving trusted others (such as family or close friends) can strengthen a person's safety network.

## Relevance and practical recommendations for allied health practice

While chiropractors and osteopaths are not mental health practitioners, the principles of relational safety, biopsychosocial care, and compassionate communication outlined in *Staying Safe from Suicide* (NHS England, 2025) have clear relevance to allied health practice. They may also have a role to play alongside other healthcare practitioners such as pharmacists, dietitians, and podiatrists [5] amongst others.

Importantly, they are not expected to assess, diagnose, or manage suicide risk. Instead, their role lies in recognising signs of distress, responding relationally, encouraging appropriate help-seeking, and documenting concerns sensitively [19].

It is recognized that this role also requires upskilling. Australian physical therapists report uncertainty or discomfort when faced with conversations about suicide or self-directed violence [20]. The same is likely true for chiropractors and osteopaths. Providing training to develop skills in asking appropriate, compassionate questions can help practitioners feel more confident and less overwhelmed in these situations. Such training also prepares practitioners to manage the personal stress that can arise when exploring sensitive disclosures. In this way, capacity building reduces reliance on rigid, standardised screening tools as a sole entry point for conversations and instead enables practitioners to engage with patients in a more authentic, relational manner.

Drawing on the guidance, chiropractors and osteopaths can adopt the following practical strategies:

### Be alert to signs of distress

Changes in mood, verbal hints of hopelessness, withdrawal from usual activities, or expressions such as "there’s no point anymore" may signal distress. These signs should prompt gentle exploration rather than be overlooked.

### Foster relational safety through empathetic engagement

Building a strong, trusting therapeutic relationship is protective. Practitioners should listen attentively, validate a person’s experiences, and maintain a non-judgmental stance. Even brief moments of compassion can have a profound impact on patients who may be feeling isolated or overwhelmed.

### Use open-ended, compassionate communication

Rather than relying on checklists or closed questions, practitioners can ask simple, open-ended questions such as: “How have you been coping lately?”, “Is there anything you’ve been finding particularly hard recently?”.

This approach reduces stigma and invites genuine conversation.

### Encourage and facilitate support-seeking

If a patient discloses significant distress, then a gentle recommendation to speak with a medical practitioner, psychologist, or crisis support service can be made. Normalising the idea of seeking help and offering practical information, such as crisis line numbers, can make a crucial difference.

### Know your local referral pathways

Being familiar with local healthcare providers, crisis services, and mental health support options allows for the provision of informed and timely guidance when patients need additional support (See Appendix 1 for some suggested regional resources)**.**

### Document sensitively and factually

Practitioners should record observations (e.g., patient statements, behaviours) objectively, avoiding assumptions about suicide risk or intent. Clear documentation protects both the person and the practitioner, ensuring continuity of care if further referral is needed.

### Care for practitioner wellbeing

Encountering patient distress can impact chiropractors and osteopaths emotionally. It is important to acknowledge these feelings and seek support through supervision, peer discussion, or professional counselling if necessary. Maintaining practitioner wellbeing sustains compassionate, effective practice over time.

While these practical recommendations provide a useful framework for identifying and responding to suicide risk, it is important to note that effective implementation requires appropriate training and skill development. Not all chiropractors and osteopaths may currently feel prepared to initiate or sustain conversations about suicide risk. Currently, there is a paucity of baseline knowledge regarding the extent to which suicide prevention competencies are embedded in chiropractic and osteopathic education. Training standards at both the entry level and within continuing professional development remain inconsistent, and evidence to support integration is limited. At least in the United States, preliminary findings show that suicide prevention training and related terminology are rarely incorporated into chiropractic curricula or licensing requirements [21]. Ongoing professional development and targeted training programs are therefore critical to ensure that practitioners are equipped to engage in these sensitive conversations safely and effectively. Strengthening the skills and confidence of health professionals through structured education and endorsed continuing professional development opportunities would help to support responsible integration of these recommendations into clinical practice.

By embedding these relational, biopsychosocial principles into everyday interactions, practitioners can help support a person’s safety and contribute meaningfully to broader suicide prevention efforts while remaining within their professional scope.

## Challenges and considerations

While chiropractors and osteopaths can meaningfully support wellbeing, engaging with people who may be experiencing suicidal distress can present real-world challenges. Recognising and preparing for these challenges is important for safe and ethical practice.

### Fear of saying the wrong thing

Many practitioners worry that asking about distress or suicide might "plant the idea" or make things worse. However, evidence consistently shows that asking sensitively does not increase risk; rather, it often provides relief and opens a pathway to support [22]. Gentle, open conversations grounded in empathy are always preferable to silence.

### Disclosure discomfort

A further challenge relates to the situational discomfort practitioners may feel when raising or responding to conversations about suicide or self-directed violence. As Hawton et al. observe, the assessment of suicide risk can be profoundly anxiety-provoking for clinicians, with many unintentionally framing questions in ways that invite a negative response before quickly moving the discussion elsewhere [11]. Such discomfort can promote avoidance and, in turn, compromise relational safety if the patient perceives hesitancy or a lack of confidence. Without adequate preparation or clear pathways for onward care, practitioners may feel ill-equipped to continue the conversation, which risks limiting disclosure. This barrier may explain why some systems have leaned on standardised screening strategies.

Addressing this requires training that builds both confidence and exposure, enabling practitioners to listen, ask compassionate, open questions and to respond in ways that support authentic engagement rather than relying solely on standardised tools. The “NHS Staying Safe” moves the focus from structured tools to the development of practitioner confidence and communication skills through education, reflective practice, and supervision.

### Uncertainty about professional boundaries

There may be a fear of overstepping the “scope of practice”. It is important to remember that practitioners are not responsible for diagnosing or treating mental illness or suicide risk. Their role is to recognise signs of distress, listen without judgement, encourage support seeking, and refer appropriately when needed. It is important that practitioners maintain professional boundaries and recognise that their role is not to fix or attempt to single-handedly support health care consumers. This may lead to an unhealthy dependency that is unsustainable for both the practitioner and clients.

Physical therapy research has found that once the disclosure of suicidal thoughts and behaviours occurred, there was a reported lack of confidence regarding role clarity and issues associated with this [20]. Further, there was a recognised need to be equipped with the knowledge, confidence, and protocols to respond effectively to suicidal distress [3]*.* It is highly likely that the same will be necessary for chiropractors and osteopaths.

### Time pressures in clinical practice

Busy clinical schedules may limit the time available for deeper conversations. However, relational safety does not require lengthy counselling sessions. Small moments of compassionate connection, such as offering a listening ear, expressing concern, and suggesting follow-up support, can be powerful even within brief appointments.

### Emotional impact on practitioners

Hearing disclosures of distress can be confronting. Chiropractors and osteopaths should acknowledge their own emotional responses and seek supervision, peer support, or professional guidance if needed. Practitioners who care for their own mental health are better equipped to care for others. This is especially true for the many practitioners who have a lived experience of mental health challenges themselves or in close relatives or friends. Whilst these experiences may enrich practitioner understanding and response, such practitioners may experience heightened emotional responses and benefit from support or supervision [23].

### Systemic barriers

Some practitioners may work in settings lacking clear referral pathways to mental health services. Proactively establishing relationships with local healthcare providers and familiarising oneself with available community resources can help bridge this gap.

Navigating these challenges requires reflection, preparation, and self-compassion. By adopting a relational, biopsychosocial mindset, chiropractors and osteopaths can confidently contribute to suicide prevention in safe and ethical ways that are consistent with their professional role.

## Conclusion

There is growing recognition of the need for a broader, system-wide public health approach that expands suicide prevention beyond traditional mental health services. As trusted members of the healthcare community, chiropractors and osteopaths can play a meaningful role in fostering safety, hope, and connection for people experiencing distress.

The recent *Staying Safe from Suicide* guidance from NHS England emphasises a shift away from unreliable risk prediction and towards relational, biopsychosocial, person-centred care. Allied Health practitioners, through the relationships they build and the holistic lens they apply, are well placed to adopt these principles in everyday practice.

By listening with compassion, recognising signs of distress, encouraging help-seeking, and collaborating within appropriate referral networks, they can contribute to a broader culture of safety and wellbeing. Small, relational acts—offered with care and authenticity—can make a difference.

In embracing this responsibility, chiropractors and osteopaths strengthen not only their connection with individual patients but also the profession’s commitment to promoting holistic health across the community.

## Supplementary Information

Below is the link to the electronic supplementary material.

Supplementary Material 1

## Data Availability

No datasets were generated or analysed during the current study.
