# Supplementary material for: Staying Safe: lessons from suicide prevention for chiropractors and osteopaths
Source: Chiropr Man Therap. 2025 Oct 21;33:47. doi: 10.1186/s12998-025-00607-x (PMC12542271; doi:10.1186/s12998-025-00607-x)
Supplement: Supplementary file 1 — Supplementary Material 1 [file 12998_2025_607_MOESM1_ESM.docx]

**Appendix 1. Resources**

**International**

International Association for Suicide Prevention / Suicidal Crisis Support <https://www.iasp.info/suicidalthoughts/>

**Australia.**

Suicide prevention: A Competency Framework For The Health Sector <https://www.suicidepreventionaust.org/wp-content/uploads/2023/09/2300905-SPA_Competency-Framework-Healthcare_v2.pdf>

Suicide Prevention: A Competency Framework

<https://www.suicidepreventionaust.org/wp-content/uploads/2021/06/Suicide-Prevention-A-Competency-Framework-2021.pdf>

**Europe**

Staying Safe From Suicide resource hub (requires you to register & log in) <https://future.nhs.uk/system/login?nextURL=%2Fconnect%2Eti%2FStayingSafefromSuicide>

**America**

American Foundation for Suicide Prevention / Best practice guidance / Get Help <https://afsp.org/get-help/>
